# Improved radiolabeling of DOTATOC with trivalent radiometals for clinical application by addition of ethanol

**DOI:** 10.1186/s41181-016-0010-8

**Published:** 2016-04-01

**Authors:** Elisabeth Eppard, Marylaine Pèrez-Malo, Frank Rösch

**Affiliations:** 1 Department of Nuclear Medicine, University Hospital Bonn, Sigmund-Freud-Strasse 25, D-53127 Bonn, Germany; 2 Institute of Nuclear Chemistry, Johannes Gutenberg-University, Fritz-Strassmann-Weg 2, D-55128 Mainz, Germany; 3 Department of Radiopharmacy, Isotopes Center, Havana, Ave. Monumental y Carr. La Rada km 31/2, Mayabeque, La Habana Cuba

**Keywords:** DOTATOC, Ethanol, Radiolabeling, Radiopharmaceuticals, Kit

## Abstract

**Background:**

Typically, metal-based radiopharmaceuticals are synthesized in aqueous solutions with no or low ethanol content. Labeling yields are defined by temperature, period of labeling, amount of precursor, pH etc. As recently observed, radiolabeling yields (RCY) seem to increase in the presence of non-aqueous solvents. Consequently, this effect was investigated systematically using ethanol as non-aqueous solvent (n-as), which is widely utilized in medicine, and DOTATOC as model compound.

**Methods:**

To determine the impact of ethanol on the radiolabeling efficacy, “standard” labeling conditions of ^68^Ga-DOTATOC (95–100 °C, 10–15 min, 20–50 μg DOTATOC, aqueous solution), i.e. 10 nmol (2.9 μM, 14.2 μg), were modified in terms of lower temperature (70 °C) to achieve lower RCY (<75 %). From those lower RCY, positive effects of increasing amounts of ethanol (0–40 vol%) could directly be observed. Labeling parameters were finally evaluated in terms of shorter reaction time and lower amount of precursor. To investigate whether the effects observed are also true for other trivalent radiometals, labeling was also performed with ^44^Sc.

**Results:**

For increasing amounts of ethanol, ^68^Ga-DOTATOC RCY at 70 °C improved significantly. RCY of ~95 % can be achieved within 10 min using 30 vol% ethanol compared to 46 % in the pure aqueous system. If “standard” temperatures of 95 °C are applied, high RCY of 89 % can be achieved within 5 min with much lower amounts of precursor, i.e. even at 0.93 nmol (0.3 μM, 1.3 μg). This also represents significantly increased specific activities. Similar behavior was observed for ^44^Sc where RCY increase successively with increasing amounts of ethanol.

**Conclusion:**

There is clear experimental evidence, that adding more than 20 vol% ethanol to the reaction mixtures significantly improve labeling efficacies. This could be demonstrated for ^68^Ga-DOTATOC and ^44^Sc-DOTATOC in terms of temperature, time and concentration of required precursor. Whether this is a principal phenomenon with practical impact on the radiopharmaceutical chemistry of trivalent metals and whether this applies to other non-aqueous solvents as well - and what the physico-chemical reasons are, remains to be studied in more detail. Nevertheless, the effect observed here will improve ^68^Ga-DOTATOC labeling and may save at least half of the usually applied amount of precursor.

## Background

Metallic radionuclides represent very important tools in nuclear medicine – both in non-invasive molecular imaging (SPECT, PET) and for radiotherapy; cf. the systematic use of e.g. ^111^In for SPECT, ^68^Ga for PET, ^90^Y and several lanthanide radioisotopes for therapy. Compared to radiopharmaceutical synthesis with non-metallic radionuclides such as e.g. ^11^C, ^18^F and other halogens, synthesis with radiometals are in most cases easier to perform and may approach kit-type protocols - as well known for ^99m^Tc, the “working horse” of nuclear medicine.

An essential part of the design of a metal-based radiopharmaceutical is the complex formation of the radioactive metal ion by ligand (or “chelate”) L. For many trivalent metals M^3+^, macrocyclic ligands, especially DOTA, show formidable thermodynamic stability, which is promising in terms of the kinetic stability of the M^3+^-L complexes once formed. This is in particular relevant in the context of molecular imaging of metal-ligand based radiopharmaceuticals to guarantee stability in vivo. However, in many cases formation rates of the M^3+^-L complexes are slow at ambient conditions. Slow formation rates are, for example, synonym to long radiolabeling protocols, to increased temperature and/or to relatively high ligand concentration. This may limit the practical use of e.g. DOTA-based radiopharmaceuticals, in particular of those labeled with short-living radionuclides like ^68^Ga (half-life t_1/2_ = 67.71 min).

Common tools to improve the efficiency of complex formation, are increased temperature (Cooper et al. 2006; De León-Rodríguez and Kovacs 2008), microwave-assisted synthesis (Pruszyński et al. 2012) or a significant excess of ligand L concentration. For example, increased temperature is commonly used to label peptidic DOTA-conjugated derivatives or DOTA-affibodies with ^111^In, ^90^Y or ^177^Lu approaching quantitative labeling efficiency at sufficient concentration of the DOTA-component (Wild et al. 2003; Laznicek et al. 2002).

Radiochemical M^3+^-L complex formation yields, in most cases, can be increased by providing a high concentration of the ligand. For short-lived radiometals, this concentration typically is at micromolar level. Depending on the kind of diagnostic or therapeutic application, however, a high specific activity of the radiopharmaceutical is preferred. For example, receptor imaging and tumor therapy require high specific activity or low concentration of the peptide, due to the limited number and affinity of transmembrane tumor receptors. Specific activity is, in this context, often given as the ratio of product radioactivity and the amount of the e.g. DOTA-precursor used. It can be significantly affected by non-radioactive metal ion contaminants competing with the desired radiometal for the ligands. These impurities are present in the radiometal stock solution, in the used chemicals and glassware. They are not necessarily reducible and result in a need for higher amounts of required precursor.

Metal-based radiopharmaceuticals are typically synthesized in aqueous solutions, where the metal ions are surrounded by strong hydration spheres. Highly charged metal ions such as Ga^3+^, Sc^3+^ and Ln^3+^ have well-defined first and second hydration shells (Lindqvist-Reis 2000). In part and among other factors, it is the power of these hydration shells, which prevents a more direct interaction of the metal cation with the donor atom of the ligand structures. It had been demonstrated a long time ago that non-aqueous solvents (e.g. isopropanol) can break up the hydrogen bond between the central metal cation and the surrounding water molecules, thereby acting as structure breaker (Idrissi and Longelin 2003). This disorder of the hydration shell in combination with preferential solvation affects the reactivity of all participants of the reaction (Pèrez-Malo Cruz and Rösch 2013; Helm and Merbach 2005; Rode et al. 2005), and in particular induces shifts in the ligands pKa values.

It was shown previously, that this effect may in particular lead to an increased efficiency of radiometal-ligand complex formation (Usacheva et al. 2012). Accordingly, a well-designed addition of a certain non-aqueous solvent to a typical aqueous solution might interestingly be used to reduce (a) reaction time, (b) temperature or (c) amount of required precursor of M^3+^-L type radiolabeling reactions.

The most attractive non-aqueous solvent in the context of medical application and radiopharmaceutical synthesis is ethanol. It is commonly used as additive and solvent in medical applications to increase the solubility of pharmaceuticals and to act as a preservative. In radiopharmaceutical chemistry, ethanol is known to inhibit ionization-induced radiolysis. As a result of these qualities, ethanol was already selected as solvent for post-processing of ^68^Ge/^68^Ga generator eluates (Eppard et al. 2014). The present report describes the effect of an extra addition of ethanol on the complex formation of ^68^Ga and ^44^Sc -based radiopharmaceuticals, utilizing DOTA-D-Phe^1^-Tyr^3^-octreotide (DOTATOC) as model compound.

The vision would be to see, whether a certain mixture of ethanol and the aqueous solution in the radiolabeling system would a) increase the labeling kinetics, b) lower the temperature required to achieve a given yield, and c) lower the amount of the ligand needed to achieve the maximum radiolabeling yields under fixed conditions. Altogether, these three effects may provide improved yields of a radiopharmaceutical like e.g. ^68^Ga-DOTATOC, currently one of the most applied DOTA-peptides for quantitative molecular imaging of neuroendocrine tumors by means of PET.

## Methods

### Experimental concept

**Fig. 1:**
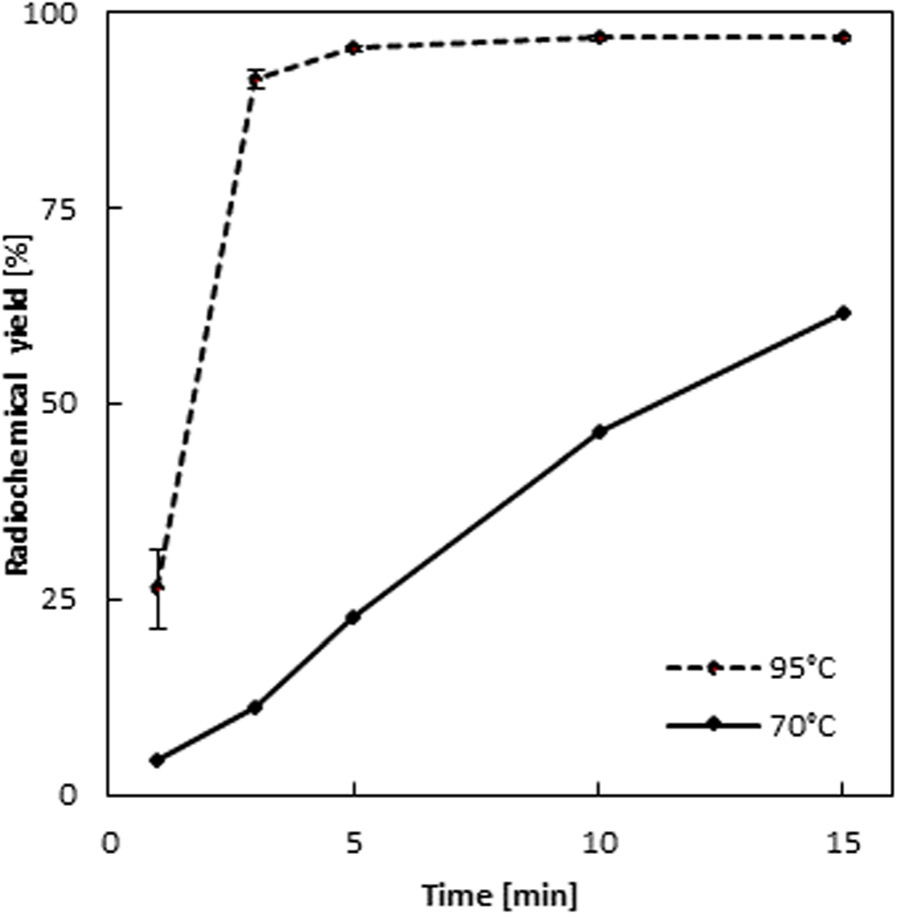
Baseline labeling kinetics of ^68^Ga-DOTATOC in pure aqueous solution at 70 and 95 °C (10 nmol/2.93 μM/14.2 μg DOTATOC, 15 min, *n* = 5)

DOTATOC was used as model compounds due to its prevalent role as therapeutic (^90^Y/^177^Lu-DOTATOC) and also diagnostic (^68^Ga-DOTATOC and ^44^Sc-DOTATOC (Pruszyński et al. 2012)) agent for neuroendocrine tumors (NET). “Standard” ^68^Ga-DOTATOC labeling is defined here by heating a pure aqueous buffer solution containing 20–50 μg DOTATOC at 95–100 °C for 10–20 min, yielding relatively high labeling yields of >95 %. In order to demonstrate the impact of ethanol on radiolabeling efficacy, those standard labeling conditions of ^68^Ga-DOTATOC were modified towards lower (~50 %) radiolabeling yield, which is obtained at lower temperature (70 °C). Indeed, this labeling profile was taken as a baseline for pure aqueous systems at 10 nmol (2.93 μM, 14.2 μg) of DOTATOC (Fig. 1). Here, ^68^Ga-DOTATOC yields are e.g. ~20 % at 5 min, ~45 % at 10 min and ~60 % at 15 min. Then, any increase of labeling yields induced by the addition of any concentration of ethanol can be determined directly at a statistically significant level. To investigate if ethanol effects only radiolabeling using ^68^Ga or also labeling with other trivalent radiometals, same experimental set-up was adopted for labeling of DOTATOC with ^44^Sc in pure aqueous solution.

### Chemicals and equipment

DOTA-D-Phe^1^-Tyr^3^-octreotide (DOTATOC) was obtained from ABX (Radeberg, Germany) and an aqueous stock solution of 1 mg/mL was prepared. All chemicals were of pure chemical grade and solvents for HPLC were obtained as HPLC grade. TraceSelect water (Sigma-Aldrich, Germany) was used in all experiments. Labeling reactions were incubated in a ThermoMixer MHR 11 (DITABIS, Digital Biomedical Imaging Systems AG).

Activity counting was determined using a borehole counter (Nuklear-Medizintechnik Dresden GmbH, Germany). RadioTLC was performed using aluminum-backed silica gel (silica-gel 60 F254; Merck, Darmstadt, Germany) and analyzed using a flatbed-scanner (Instant Imager®, Packard Canberra, Schwadorf, Austria). HPLC was performed using Hitachi L-7100 pump system coupled with UV (Hitachi L-7400) and radiometric (Gaby Star, Raytest Isotopenmessgeräte, GmbH, Straubenhardt, Germany) detectors.

### Labeling with ^68^Ga

^68^Ga obtained from a 1.100 MBq ^68^Ge/^68^Ga generator (Eckert & Ziegler Strahlen- und Medizintechnik AG, Berlin, Germany) with TiO_2_ matrix, was eluted with 5 mL 0.1 N HCl and post-processed following a literature procedure (Zhernosekov et al. 2007). Labeling of DOTATOC was performed by adding the acetone post-processed ^68^Ga eluate (400 μL) to a solution of DOTATOC (10 nmoL; 2.93 μM, 14.2 μg) in 3 mL water containing 0–40 vol% ethanol. The reaction mixture was stirred at 70 °C and aliquots were taken at 1, 3, 5, 10 and 15 min to analyze radiolabeling kinetics.

All experiments were performed on a laboratory scale and repeated three times (*n* = 3).

In order to compare the labeling yields of the standard labeling protocol at 95 °C with and without ethanol content, the effect of ethanol addition (40 vol%) on labeling yields was evaluated with varying concentration of peptide (00.003–31 nmol/0.001–10 μM/0.0043–44.1 μg).

### Labeling with ^68^Ga on a module system

Finally, experiments with ^68^Ga-DOTATOC were performed in addition using a module system to evaluate the impact of ethanol on labeling efficacy with regard to routine tracer production (Eckert & Ziegler, Modular Lab Eazy).

^68^Ga obtained from a 1.100 MBq ^68^Ge/^68^Ga generator (Eckert & Ziegler Strahlen- und Medizintechnik AG, Berlin, Germany) with TiO_2_ matrix, was eluted with 0.1 N HCl and post-processed with ethanol/HCl solution according literature (Eppard et al. 2014; Zhernosekov et al. 2007). In order to achieve a 40 vol% ethanol/water mixture, there are two options, which is either offline or online. Offline means the preparation of the mixture by dissolving the needed amount of DOTATOC in water or aqueous buffer and subsequent adding 40 vol% of ethanol. To this mixture the purified fraction of ^68^Ga is added before heating. In contrast, online corresponds to the preparation of the “standard” aqueous solution containing DOTATOC, while the post-processed ^68^Ga added already contains the needed amount of ethanol. This is achieved by purifying ^68^Ga eluates from ^68^Ge generators utilizing a modified version of the cation exchange-based post-processing introduced in 2007 (Zhernosekov et al. 2007) according to a recently published variant based on ethanol/HCl mixtures to desorb ^68^Ga from the cation exchanger resin (Eppard et al. 2014).

Labeling of DOTATOC was performed on a radiosynthesis system (Modular-Lab eazy, Eckert & Ziegler Strahlen- und Medizintechnik AG, Berlin, Germany) and constant reaction time (480 s) by adding aliquots of DOTATOC stock solution (1 mg/mL) to mixtures of post-processed ^68^Ga eluate (1 mL) and 1 M NH_4_OAc solution (900 μL) which corresponds to an ethanol content of 47 vol%. The concentration of DOTATOC was varied between 7.0 nmol (3.67 μM, 10 μg) and 28.1 nmol (14.56 μM, 40 μg) with constant temperature (110 °C as defined by the module).

### Labeling with ^44^Sc

^44^Sc was eluted from a 180 MBq ^44^Ti/^44^Sc generator using a 0.005 M H_2_C_2_O_4_/0.07 M HCl solution (Filosofov et al. 2010) and post-processed as previously described (Pruszyński et al. 2012). The purified aqueous eluate was used for further labeling studies with DOTATOC (20 nmol, 6.60 μM, 28.4 μg) and varying ethanol content (0–40 vol%). The reaction mixture was stirred at 70 °C for 30 min and aliquots were taken at 1, 3, 5, 10, 20 and 30 min to analyze radiochemical yields.

### Quality control

RadioTLC was used to analyze the reaction yields. Aliquots of each reaction solution were spotted on TLC-plates, developed with 0.1 M sodium citrate solution (pH 4.0) and analyzed using a flatbed-scanner. Additionally, radiochemical purity was analyzed using radioHPLC with a LiChrosphere 100 RP-18 EC column (5 μm, 250 × 4 mm). The gradient elution system utilized mobile phase A (water + 0.01 % TFA) and mobile phase B (acetonitrile + 0.01 % TFA). A flow rate of 0.8 mL/min was used, starting with 82 % A for 2 min; then the gradient was increased to 30 % B during next 25 min and then held at 30 % B for 6 min. Afterwards gradient parameters returned to the initial conditions during 12 min.

## Results

### ^68^Ga-DOTATOC: Labeling yields

The effect of adding ethanol was first investigated for radiolabeling DOTATOC, using 10 nmol (2.93 μM) DOTATOC, at 70°. Indeed, this labeling profile was taken as a reference for pure aqueous systems at 10 nmol (2.93 μM, 14.2 μg) of DOTATOC (Fig. 1). Then, any effect on labeling yields induced by the addition of any concentration of ethanol can be determined directly at a statistically significant level.

**Fig. 2:**
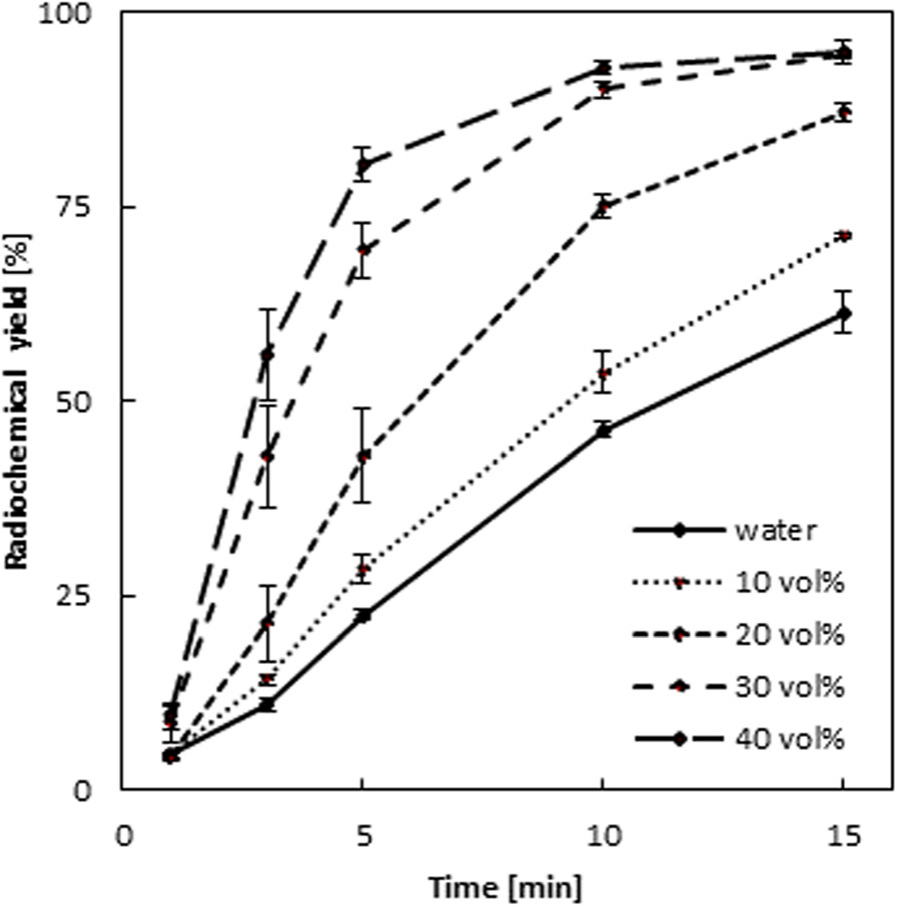
Labeling kinetics of ^68^Ga-DOTATOC in presence of 0–40 vol% ethanol (10 nmol/2.93 μM/14.2 μg DOTATOC, 70 °C, *n* = 3)

While addition of 10 vol% ethanol after 15 min leads to a small but highly reproducible increase by factor of 1.2, a further increase of the ethanol content up to 40 vol% results in labeling yields of approximately 95 % within 15 min, which is an increase by a factor of 1.5 at 70 °C for DOTATOC. These impressive effects are illustrated in Fig. 2 for the labelling yields for the 4 different concentrations of ethanol. The relative increase of ^68^Ga-DOTATOC labeling yields in the ethanol system (relative to pure aqueous solution) observed at 15 min reaction time is 1.2 (10 vol%), 1.4 (20 vol%), 1.5 (30 vol%) and 1.5 (40 vol%) at 70 °C.

### ^68^Ga-DOTATOC: Labeling kinetics

**Fig. 3:**
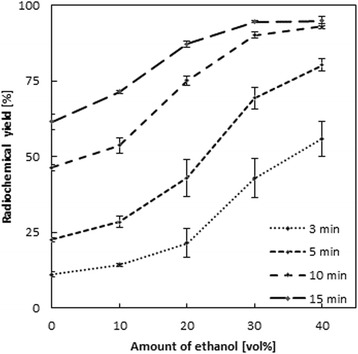
Increase of labeling yield of ^68^Ga-DOTATOC depending on the individual amounts of additional ethanol (10 nmol/2.93 μM/14.2 μg DOTATOC, 70 °C, *n* = 3)

Interestingly, the effect is even more pronounced at shorter reaction times. Figure 3 illustrates this effect.

The observed relative increase at e.g. 5 min is 3.6 for 40 vol% (at 70 °C). Table 1 gives the relative increase of the yields for 5, 10 and 15 min reaction period for 10–40 vol% of ethanol compared to the pure aqueous system.

**Table 1:** Increase of ^68^Ga-DOTATOC labeling yields depending on the reaction period by factor. The dramatic increase for short reaction periods such as e.g. 5 min is highlighted in grey

Time [min]	Amount of ethanol in the reaction mixture [vol%]				
	0	10	20	30	40
3	1	1.3	2.0	3.9	5.1
5	1	1.3	1.9	3.1	3.6
10	1	1.2	1.6	1.9	2.0
15	1	1.2	1.4	1.5	1.5

### ^68^Ga-DOTATOC: Amount of DOTATOC

With the dramatic increase in reaction yields in ethanol containing solvent mixtures, in particular at shorter reaction periods, one may stipulate, that ^68^Ga-DOTATOC labeling is more effective with lower amounts of DOTATOC in ethanol/water mixtures compared to the standard aqueous system (with “standard” here again referring to pure aqueous solutions and reaction temperatures of 95 °C). Consequently, the dependence of the radiochemical yield on the concentration of DOTATOC in presence of 40 vol% ethanol was investigated and compared to identical non-ethanol systems for synthesis temperatures of 95 °C. The reaction time was 5 min in both cases.

**Fig. 4:**
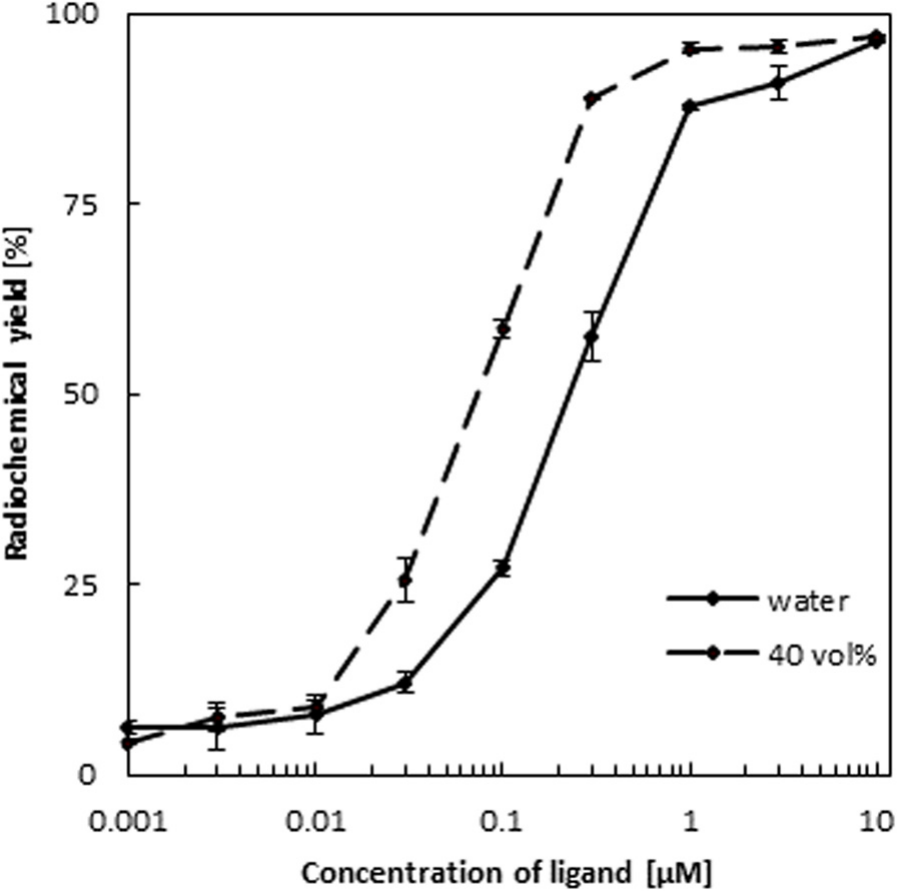
Dependence of the radiochemical yield of ^68^Ga-DOTATOC on the concentration of DOTATOC in presence of 40 vol% ethanol (0.003–31 nmol/0.001–10 μM/0.0043–44.1 μg DOTATOC, 95 °C, 5 min, *n* = 3)

Figure 4 shows the effect of 40 vol% ethanol in the reaction mixture on labeling yields with the DOTATOC precursor amount varied from 0.003 to 31 nmol (0.001–10 μM). For high amounts such as 31 nmol (10 μM) DOTATOC, the radiochemical yields are similar (96.3 %/97 %) for the pure aqueous and the ethanol/water system. However, in the pure aqueous system the radiochemical yields drop with decreasing amount of DOTATOC, reaching values of 90.7 ± 2.2 %, 87.7 ± 0.3 % and 57.5 ± 0.3 % at 9.3 nmol (3 μM), 3.1 nmol (1 μM) and 0.9 nmol (0.3 μM) DOTATOC, respectively. In contrast, the presence of 40 vol% ethanol keeps ^68^Ga-DOTATOC labeling still efficient with labeling yields of 95.6 ± 0.9 %, 95.4 ± 0.6 % and 88.7 ± 0.2 % for the same concentrations of DOTATOC. Notably, 3.1 nmol (1 μM) of DOTATOC thus guarantee ^68^Ga-DOTATOC yields of >95 %, meeting the requirement of the European Pharmacopoeia on the product purity of ^68^Ga-DOTATOC for i.v. injections. In pure aqueous systems, this >95 % level is achieved at one order of magnitude higher concentration of DOTATOC only.

A significant effect of ethanol on labeling efficacies occurs in solutions with concentrations between 0.03 and 3 μM. The highest increase can be observed at 0.1 μM (0.3 nmol) DOTATOC, where labeling yields are larger by a factor of 2.2 in the ethanol/water system.

### ^68^Ga-DOTATOC: Specific activity

The radiolabeling yields > 95 % with only 1 μM DOTATOC solutions obtained in 40 vol% ethanol mixtures after 5 min instead of 10 μM in aqueous “standard” systems are also of practical interest. Compared to the pure aqueous system, the same labeling yields are achieved at a 10fold lower concentration of DOTATOC. This directly increases the specific activity of ^68^Ga-DOTATOC (i.e. the radioactivity of ^68^Ga relative to the amount of the DOTATOC) by a factor of 10.

### ^68^Ga-DOTATOC: Labeling on an automated module

Once the effect of adding ethanol to the reaction mixtures for ^68^Ga-DOTATOC was demonstrated at laboratory scale, the next challenge was to transfer this successful method to automated synthesis modules. To facilitate the daily work with ^68^Ga, but also ^177^Lu and ^90^Y, Eckert & Ziegler has developed a radiosynthesis system which was used in this study. The cassette based synthesizer works with a technology which uses pressure differences to transfer the liquids between the reaction vessels and pre-defined software templates with easy-to-operate user interface guarantee high yields, radiochemical purity as well as a short synthesis time.

**Fig. 5:**
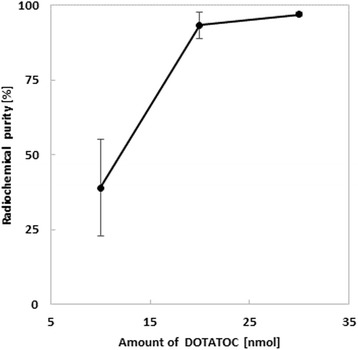
Radiochemical purity of ^68^Ga-DOTATOC depending on the amount of precursor using the automated labeling module ModularLab eazy (t = 480 s, T = 110 °C, 900 μL NH_4_OAc buffer pH 7, 1 ml N5, *n* = 3)

The amount of ethanol in the reaction mixture accounts to 42 vol% under the conditions used. An amount of precursor of up to 30 μg (21 nmol, 0.01 μM) leads to radiolabeling yields > 95 %. Even with low amounts of precursor (20 μg, 14 nmol, 0.007 μM) radiolabeling yields > 90 % can be obtained. It should be noted, that ^68^Ga-DOTATOC labeling protocols supplied by most of the commercial manufacturers recommend the use of more than 30 μg (21 nmol, 0.01 μM) of DOTATOC Fig. 5.

### ^44^Sc-DOTATOC: Labeling yields

With these dramatic effects observed for ^68^Ga-DOTATOC, a logic question was to verify whether the effect observed is specific for the ^68^Ga-radiocation, or whether it applies to other trivalent metals used in nuclear medicine as well. Therefore DOTATOC was labeled with the generator-derived positron emitter ^44^Sc. In the case of ^44^Sc, high labeling yields of ^44^Sc-DOTATOC in pure aqueous systems containing ammonium acetate buffer have been described earlier for 95 °C temperature, but somewhat extended reaction periods compared to ^68^Ga-DOTATOC (Pruszyński et al. 2012).

**Fig. 6:**
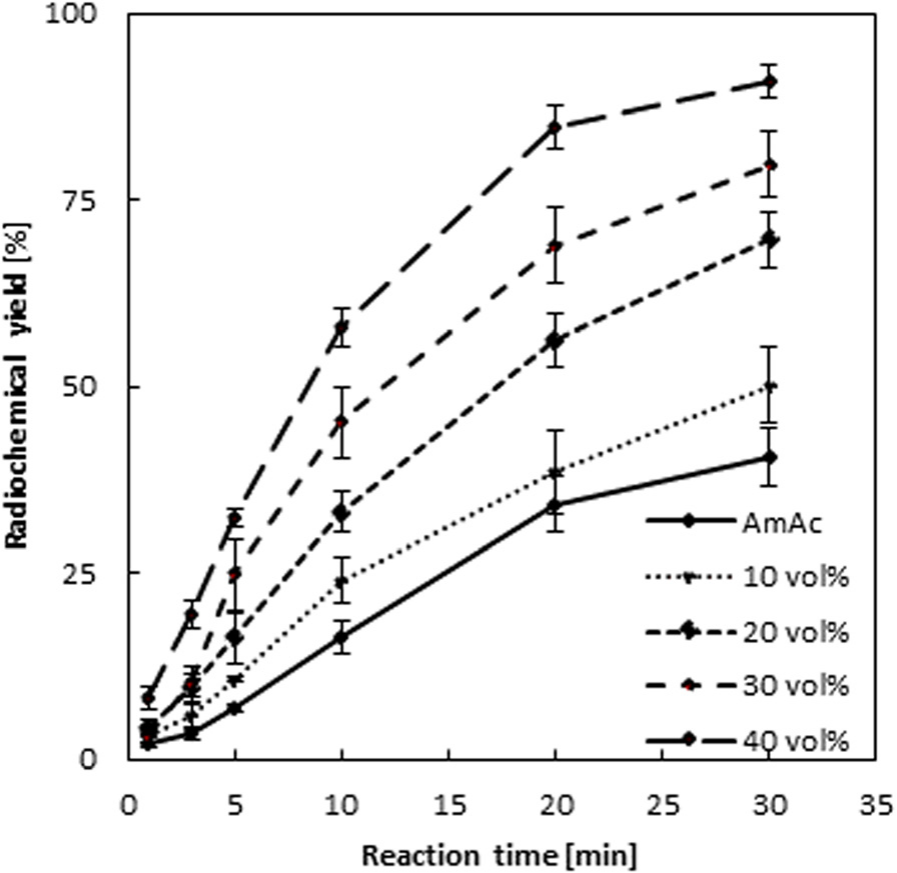
Labeling kinetics of ^44^Sc-DOTATOC in presence of 10–40 vol% ethanol compared to the pure aqueous solution containing ammonium acetate (AmAc) as buffer (20 nmol/6.62 μM/28.5 μg DOTATOC, 70 °C, *n* = 3)

Indeed, a similar behavior was observed for ^44^Sc-DOTATOC. Radiochemical yields increase successively with increasing amounts of ethanol but not that rapidly like for ^68^Ga-DOTATOC. At 40 vol% of ethanol, labeling yields are more than double (by factor 2.2) after 20 and 30 min (Fig. 6). Under the same conditions of 70 °C and 30 min, yields of > 90 % can be obtained using 40 vol% ethanol compared to 40.7 ± 3.9 % in the ethanol-free system. Also similar to ^68^Ga-DOTATOC, the relative increase is even more pronounced for short reaction periods (Pruszyński et al. 2012; Flowers L et al. 2003).

## Discussion

The influence of a solvent on solvation and complex formation equilibria of stable metal ions and ligands is a known phenomenon (Helm and Merbach 2005). For example, preferential solvation may occur when the solution is a mixture of two or more solvents, and effects the reactivity of all participants specifically (Helm and Merbach 2005; Rode et al. 2005; Usacheva et al. 2012). This phenomenon, however, was never applied in radiopharmaceutical synthesis to economize effectively metal-based radiolabeling. The results shown here for labeling of DOTATOC with the trivalent positron emitters ^68^Ga and ^44^Sc are dramatic. The phenomenon is of interest from a “pure” scientific point of view as it appears to be much more pronounced than in conventional, e.g. non-radio-chemistry. In parallel, it may be relevant to the routine synthesis of radiopharmaceuticals based on these metallic radionuclides. The main features are summarized using ^68^Ga-DOTATOC as a proof-of-principle PET-radiopharmaceutical. There are at least 5 basic factors to discuss.

### Radiochemical labeling yields

For a given reaction system and temperature, the addition of ethanol increases the radiochemical labeling yields depending on the vol% of the non-aqueous solvent and the reaction period. For example, after 3 min the increases are as much as 130–500 % (or factors of >1.3–5) compared to yields achieved with the ethanol-free, pure aqueous system. At 40 vol% the effect appears to approach saturation.

### Reaction kinetics

For a given reaction system and temperature, ethanol facilitates the reaction kinetics. Depending on the vol% of the non-aqueous solvent, similar yields, compared to the ethanol-free system, are obtained faster by factors of >2 or even >5. This is in particular relevant for short-living radionuclides such as ^68^Ga.

### Amount of precursor

For a given reaction system, temperature and period, the addition of ethanol increases the reaction yields for identical amounts of precursor (e.g. DOTATOC). Depending on the vol% of the non-aqueous solvent, it can reduce the amount of DOTATOC needed to e.g. achieve radiochemical yields > 95 % by a factor of 10 or more. This is synonym to an increase of the ^68^Ga-DOTATOC product specific activity by a factor of 10 or more, which is relevant especially in context of receptor imaging and therapy.

### Duration of synthesis

Applying optimum reaction conditions, one might guarantee ^68^Ga-DOTATOC constant labeling yields > 98 % in routine synthesis. This is well above the current recommendation of a monograph of the European Pharmacopoeia (European pharmacopoeia 2013). In this case, radiochemical purification procedures are not needed anymore. In practice, the duration of the synthesis from elution of the generator until formulation of the final product will be reduced due to deletion of the purification step. Effectively, this also contributes to an increase in final product activity. In particular this is relevant for short-living radionuclides such as ^68^Ga: saving 10 min of reaction time saves almost 11 % of radioactivity – i.e. increases the final product activity of e.g. ^68^Ga-DOTATOC by 11 %.

### Automated modules

This method based on ethanol/HCl mixtures ^68^Ga is adsorbed from the initial generator eluate online, and subsequently releases the purified ^68^Ga, using solutions in which the acetone from the original cation exchange post-processing is replaced by ethanol. The post-processing includes a washing step using 1 mL 80 % EtOH/0.15 M HCl and the elution step using 1 mL 90 % EtOH/0.9 M HCl. The steps involved are very similar to those of the acetone-based method, which has been successfully incorporated into EZAG® modules. The method is very effective with regard to the removal of radiochemical (^68^Ge) and chemical (Fe, Ti) impurities which perturb radiolabeling. The processed ^68^Ga eluate facilitates high labeling yields and specific activities due to the increased chemical and radiochemical purity. In contrast to the acetone-based protocol, the purified eluate resulting from the ethanol method is more acidic and needs buffer formulation for radiolabeling. The positive effect of ethanol on radiolabeling yields can be implemented easily on automated module systems taking the advantage of ethanol-based cation-exchange post-processing. Legal requirements can already be fulfilled without purification using 30 μg (21 nmol, 0.01 μM) precursor, which results in reduced duration of synthesis and higher specific activity. This is significantly lowered compared to the amount recommended by commercial suppliers for reliable synthesis on automated modules.

In the present study, the experimental effects of adding various amounts of ethanol to the “standard” aqueous solutions in the preparation of ^68^Ga-DOTATOC are evident. From a radiopharmaceutical point of view, this relates to a dramatic increase of the efficacy of ^68^Ga-DOTATOC labeling reactions. It concerns three experimental parameters rather directly, namely (1) labeling yields, which increase compared to yields in the ethanol-free, pure aqueous system, (2) labeling kinetics, which are faster and (3) amounts of DOTATOC precursor needed, which are less by at least an order of magnitude. Altogether, it (4) allows robust and reliable ^68^Ga-DOTATOC batch purities of > 95 % as determined both by radioTLC and radioHPLC. There was no evidence of any colloid formation in the ethanol-containing solution even when aiming high specific activities. However, there are even four more effects, which result from the ones mentioned. One is the significant increase in specific activity of ^68^Ga-DOTATOC preparations as a consequence of (3).

A further effect is on lyophilized kit-type samples of DOTATOC which may be used, where the adequate vol% of ethanol is added immediately prior to the addition of the purified ^68^Ga generator eluate. This may further stimulate the development of kit-type formulations of ^68^Ga radiopharmaceuticals. In the case of ^68^Ga-DOTATOC, the high labeling yield of > 95 % makes purification steps obsolete, and the formulation just diluted with saline followed by sterile filtration is ready for application. This would make ^68^Ga-DOTATOC synthesis approaching the ease of ^99m^Tc-kit preparations. Moreover, those reaction protocols are easily to adopt on automated or semi-automated modules available for radiometal-based synthesis. The increased yield at shorter reaction periods and the avoiding of any additional purification step may cumulatively save 10 min or more until the ^68^Ga-DOTATOC batch is ready for injection. This increases the real ^68^Ga-DOTATOC activity available by about 10 % or more. Finally, there is another effect of rather economic value: an institution routinely performing ^68^Ga-DOTATOC PET for neuroendocrine tumors may, depending on the frequency of batch synthesis, need e.g. 100 μg of DOTATOC each day. Within 100 working days, which maybe is the shelf-life of a ^68^Ge/^68^Ga generator, this accumulates to 10 mg. Nevertheless, with saving the amount of DOTATOC, there comes a significant economic impact.

## Conclusion

The authors hypothesize, that this may constitute a fundamental aspect in radiopharmacy. The effects shown for ^68^Ga-DOTATOC are not unique for this particular radiopharmaceutical. It holds true for ^44^Sc also, and underlines the assumption, that the effect achieved by adding ethanol to pure aqueous reaction mixtures facilitates complex formation in many directions in general: Mixtures of aqueous and ethanol significantly improve labeling efficacies of metal-based radiopharmaceuticals e.g. ^68^Ga-DOTATOC in terms of temperature, time and concentration of required precursor (Pèrez-Malo Cruz and Rösch 2013).

However, it’s scientific rational and its applicability to various radiometals of varying valence states requires much more and systematic investigation, which are currently performed. Simultaneously, the physico-chemical nature of the impact of ethanol/water mixtures or mixtures using other non-aqueous solvents on the radiometal-ligand complex formation deserves further scientific investigation. This is beyond the scope of the present study, which intends to highlight the practical impact for radiopharmaceutical chemistry only. Those studies are ongoing.

### Compliance with ethical standards

Ethical approval: This article does not contain any studies with human participants or animals performed by any of the authors.
